# Age of the Domestic Animals

**Published:** 1892-09

**Authors:** 


*Age of the Domestic Animals.* By RUSH SHIPPEN HUIDEKOPER, M.D. Pp. viii., 217. Philadelphia: F. A. DAVIS. 1891.

Though of more usefulness to the practitioner of veterinary than of human medicine, this book, nevertheless, is of high scientific interest as well as practical value. Treating most at length of the horse, but more briefly also of the human and other domesticated mammals, the work explains how the age of the various creatures may be determined by the teeth and other points. The bibliography connotes how imperfect hitherto has been such information in the English language.

